# Intraoperative MET-receptor targeted fluorescent imaging and spectroscopy for lymph node detection in papillary thyroid cancer: novel diagnostic tools for more selective central lymph node compartment dissection

**DOI:** 10.1007/s00259-022-05763-3

**Published:** 2022-04-07

**Authors:** Pascal K. C. Jonker, Madelon J. H. Metman, Luc H. J. Sondorp, Mark S. Sywak, Anthony J. Gill, Liesbeth Jansen, Thera P. Links, Paul J. van Diest, Tessa M. van Ginhoven, Clemens W. G. M. Löwik, Anh H. Nguyen, Robert P. Coppes, Dominic J. Robinson, Gooitzen M. van Dam, Bettien M. van Hemel, Rudolf S. N. Fehrmann, Schelto Kruijff

**Affiliations:** 1 Department of Surgery, University Medical Center Groningen, University of Groningen, Hanzeplein 1, Groningen, 9713 GZ the Netherlands; 2 Department of Endocrine Surgery and Surgical Oncology, Royal North Shore Hospital, St Leonards, Australia; 3 Department of Biomedical Sciences of Cell & Systems – Section Molecular Cell Biology, University Medical Center Groningen, University of Groningen, Groningen, the Netherlands; 4 Department of Anatomical Pathology, NSW Health Pathology, Royal North Shore Hospital, St Leonards, Australia; 5 Sydney Medical School, University of Sydney, Sydney, Australia; 6 Cancer Diagnosis and Pathology Group, Kolling Institute of Medical Research, Royal North Shore Hospital, St Leonards, Australia; 7 Department of Endocrinology, University Medical Center Groningen, University of Groningen, Groningen, the Netherlands; 8 Department of Pathology, University Medical Center Utrecht, Utrecht, the Netherlands; 9 Sidney Kimmel Comprehensive Cancer Center, Johns Hopkins, Baltimore, MD USA; 10 Department of Surgery, Erasmus MC Cancer Institute, Rotterdam, the Netherlands; 11 Department of Radiology and Nuclear Medicine, Erasmus MC, Rotterdam, the Netherlands; 12 Department of Pathology, Erasmus MC, Rotterdam, the Netherlands; 13 Department of Radiation Oncology, University Medical Center Groningen, University of Groningen, Groningen, the Netherlands; 14 Department of Otorhinolaryngology and Head and Neck Surgery, Erasmus MC Cancer Institute, Rotterdam, the Netherlands; 15 Department of Nuclear Medicine and Molecular Imaging, University Medical Center Groningen, University of Groningen, Groningen, the Netherlands; 16 AxelaRx/TRACER B.V, Groningen, the Netherlands; 17 Department of Pathology, University Medical Center Groningen, University of Groningen, Groningen, the Netherlands; 18 Department of Medical Oncology, University Medical Center Groningen, University of Groningen, Groningen, the Netherlands

**Keywords:** Papillary thyroid cancer, Molecular fluorescence-guided imaging, Spectroscopy, Lymph node imaging

## Abstract

**Purpose:**

Patients undergoing prophylactic central compartment dissection (PCLND) for papillary thyroid cancer (PTC) are often overtreated. This study aimed to determine if molecular fluorescence-guided imaging (MFGI) and spectroscopy can be useful for detecting PTC nodal metastases (NM) and to identify negative central compartments intraoperatively.

**Methods:**

We used a data-driven prioritization strategy based on transcriptomic profiles of 97 primary PTCs and 80 normal thyroid tissues (NTT) to identify tumor-specific antigens for a clinically available near-infrared fluorescent tracer. Protein expression of the top prioritized antigen was immunohistochemically validated with a tissue microarray containing primary PTC (*n* = 741) and NTT (*n* = 108). Staining intensity was correlated with 10-year locoregional recurrence-free survival (LRFS). A phase 1 study (NCT03470259) with EMI-137, targeting MET, was conducted to evaluate safety, optimal dosage for detecting PTC NM with MFGI, feasibility of NM detection with quantitative fiber-optic spectroscopy, and selective binding of EMI-137 for MET.

**Results:**

MET was selected as the most promising antigen. A worse LRFS was observed in patients with positive versus negative MET staining (81.9% versus 93.2%; *p* = 0.02). In 19 patients, no adverse events related to EMI-137 occurred. 0.13 mg/kg EMI-137 was selected as optimal dosage for differentiating NM from normal lymph nodes using MFGI (*p* < 0.0001) and spectroscopy (*p* < 0.0001). MFGI identified 5/19 levels (26.3%) without NM. EMI-137 binds selectively to MET.

**Conclusion:**

MET is overexpressed in PTC and associated with increased locoregional recurrence rates. Perioperative administration of EMI-137 is safe and facilitates NM detection using MFGI and spectroscopy, potentially reducing the number of negative PCLNDs with more than 25%.

**Clinical trial registration.:**

NCT03470259.

**Supplementary Information:**

The online version contains supplementary material available at 10.1007/s00259-022-05763-3.

## Introduction

Papillary thyroid cancer (PTC) is an indolent malignancy representing 90% of novel thyroid cancer cases. Curative treatment, consisting of thyroidectomy combined with postoperative radioactive iodine therapy, results in 10-year survival rates exceeding 90% [1]. The standard imaging modality for the central compartment is preoperative ultrasound, staging of 50 to 70% of the PTC patients with a cN0 nodal status [2–4]. However, only 50% of the patients staged with a negative nodal status undergoing prophylactic central compartment lymph node dissection (CLND) are true negative on final histopathology [5]. Due to the presence of lymph node metastases in PTC patients with a cN0 nodal status, the value of a standard prophylactic CLND is debated. Although prophylactic CLND does not improve survival, proponents argue that it facilitates more accurate risk stratification and reduces recurrence rates, thus avoiding challenging re-operations associated with more postoperative complications. Critics point out that most patients undergoing prophylactic CLND are overtreated: 20 prophylactic CLNDs are needed to prevent one local recurrence needing re-operation [6]. Moreover, prophylactic CLND exposes many patients to unnecessary morbidities, such as recurrent laryngeal nerve palsy and permanent hypoparathyroidism, negatively impacting quality of life [7]. As visual and tactile inspection during surgery is unreliable to rule out nodal metastases, an intraoperative diagnostic tool with a high negative predictive value (NPV) could provide surgeons with real-time information about the nodal stage of the central compartment. Such a tool could improve the selection of PTC patients with a true negative central compartment, ultimately avoiding a prophylactic CLND and decreasing morbidity.

Multiple clinical studies have demonstrated that molecular fluorescence-guided imaging (MFGI) and quantitative multidiameter single-fiber reflectance and single-fiber fluorescence (MDSFR/SFF) spectroscopy of near-infrared fluorescent (NIRF) tracers are safe tools for in vivo tumor detection [7]. In patients with head and neck squamous cell carcinoma (HNSCC) who underwent neck dissection, MFGI alone showed a negative predictive value of 99% for lymph node metastases [8]. Based on these findings, we hypothesized that MFGI and quantitative spectroscopy can help rule out the presence of nodal metastases in the central compartment during PTC-related thyroid surgery and assist in real-time intraoperative clinical decision-making.

In this study, we initially focused on PTC-specific target selection by identifying the highest overexpressed gene in primary PTC tumors with a downstream protein product targeted by a clinically available NIRF tracer. We then determined the association between positive immunohistochemical staining status of the selected protein target in primary PTC tumors with 10-year locoregional recurrence rates in a large international multicenter clinical dataset. Subsequently, we performed in vitro experiments to assess the specific binding of the selected NIRF tracer (EMI-137) to its target (the receptor tyrosine kinase MET). The proto-oncogene *MET* encodes the plasma membrane localized tyrosine-kinase receptor MET, which is activated by the ligand hepatocyte growth factor (HGF) [9, 10]. Several factors may contribute to MET overexpression in PTC. These include dysregulation or mutation of oncogenes (*RAS*, *ETC*, *RET*, and *BRAF*), downregulation of *MET* suppressing microRNAs, or autocrine stimulatory loop of the HGF/MET axis activated by PTC cells [11–17]. Activation of MET causes different cellular responses such as proliferation of the rat sarcoma (RAS) effector, scattering effects through the phosphatidylinositol 3-kinase (PI3K) pathway, and morphogenetic effects mediated by STAT3 signal transducers [18–20]. HGF binding leads to rapid internalization of MET and its accumulation near the nucleus. Following internalization and traffic to the perinuclear endomembrane, MET sustains activity of previously engaged pathways such as Rac1 [10]. These combined biological responses following MET activation may facilitate PTC tumor invasiveness and nodal metastasis. Following the selection of MET as target antigen, we then conducted a first-in-human clinical study to determine the perioperative safety profile of EMI-137 and optimal dosage to detect PTC nodal metastases using fluorescence epi-illumination. The acquired imaging data demonstrated the potential clinical relevance of MFGI for detecting true negative central compartments. Additionally, we tested the feasibility of using MDSFR/SFF spectroscopy to quantify fluorescence in PTC nodal metastases. In the final part of the clinical study, we validated our results by assessing the specific binding of EMI-137 in the PTC lymph node metastases and normal lymph nodes of the included PTC patients.

## Methods

**Fig. 1:**
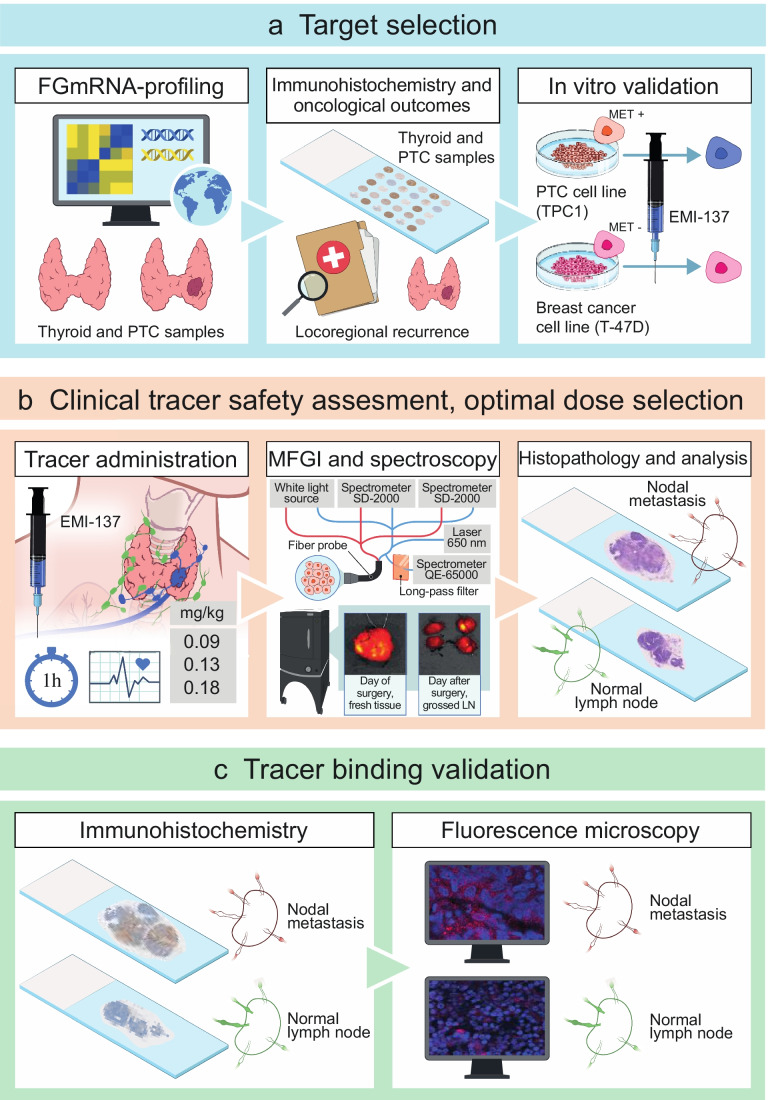
Study workflow illustrating each of the steps for target selection (**a**), clinical tracer safety assessment and optimal dosage selection (**b**), and validation of in vivo tracer binding following intravenous administration (**c**). Abbreviations: EMI-137, investigational medicinal product; FGmRNA-profling, functional genomic mRNA profiling; h, hour; IHC, immunohistochemistry; mg, milligram; MFGI, molecular fluorescence-guided imaging; kg, kilogram; LN, lymph nodes; NLN, normal lymph nodes; NM, nodal metastases; PTC, papillary thyroid cancer

This section contains a concise version of the methods used in this study. More details are provided in the supplementary information. The study workflow is depicted in Fig. 1.

### Target selection

The acquisition and processing of microarray expression data of primary PTC (*n* = 97) and normal thyroid tissue (*n* = 80) have been described previously and are summarized in the supplementary information [21]. To capture the downstream effects of genomic alterations at gene expression levels, a class comparison between PTC and normal thyroid tissue was performed using functional genomic mRNA profiling (FGmRNA-profiling), as described elsewhere [22]. The list of significantly upregulated genes in PTC was compared with the targets of 12 clinically available NIRF tracers used for MFGI [7]. The highest-ranked overexpressed gene targeted by a clinically available NIRF tracer was selected to assess downstream protein expression.

Following approval of the Northern Sydney Local Health District Human Research Ethics Committee, the protein expression of the candidate gene was validated by immunohistochemical staining using a MET-targeting antibody (Cell Signaling Technology Cat# 8198, Danvers, USA RRID:AB_10858224), on a tissue microarray (TMA) with tissue cores acquired from primary PTC tumors (741 cases) and normal thyroid tissue (108 cases). An expert endocrine pathologist quantified staining intensities by calculating an H-score per tissue type per patient based on the percentage of cells (0–100%) for each staining intensity (0, 1 + , 2 + , 3 +) from available cores. A *H*-score ≥ 150 was considered as positive staining. Patient demographics, tumor characteristics, treatment details, 10-year locoregional recurrence-free survival (LRFS), and overall survival (OS) of tumors with positive and negative MET staining status were compared. In addition to MET expression status of the primary PTC tumors, previously identified risk factors for locoregional recurrence (nodal metastasis (pN1 vs c/pN0), tumor size (≥ 40 mm vs < 40 mm), multifocality (multifocal vs unifocal), extrathyroidal extension (present vs absent), and vascular invasion (present vs absent)) were used as input variables for a multivariate analysis using the Cox proportional-hazards model. [23, 24].

In vitro experiments were performed to assess specific binding of the selected NIRF tracer (EMI-137) in a PTC cell line (TPC1, RRID:CVCL_6298) with mRNA overexpression of the chosen/candidate target (MET) and a negative control breast cancer cell line (T47D, RRID:CVCL_0553).

### Clinical tracer safety assessment and optimal dosage selection

#### Study design

After the target selection process, a multicenter, phase 1 dose-escalation study of EMI-137 (Edinburgh Molecular Imaging Ltd, Edinburgh, UK)—a fluorescent-labeled peptide targeting the MET receptor—was performed between June 2018 and December 2019 to assess tracer safety, feasibility, and optimal dosage for the detection of PTC nodal metastases. The study was approved by the local medical ethical committees, registered at ClinicalTrials.gov (NCT03470259), and conducted according to the Declaration of Helsinki (Fortaleza, Brazil, 2013 amendment) at the University Medical Center Groningen (UMCG) and the Erasmus University Medical Center (EMC). Patients older than 18 years, with preoperatively confirmed PTC (Bethesda VI) by fine-needle aspiration and eligible to undergo a lymph node dissection for primary or recurrent disease, were eligible for inclusion. Written and signed informed consent was obtained. Based on the calculated half-life of 2 h and 30 min, patients were injected 2 h before surgery with an intravenous bolus injection of 0.09 mg/kg, 0.13 mg/kg, or 0.18 mg/kg EMI-137 (peak excitation and emission at 653 nm and 675 nm) [25]. The surgical procedure was performed per standard of care. Patients were observed for 1 h following injection to record vital parameters. Adverse events were recorded according to the National Cancer Institute Common Terminology Criteria for Adverse Events (CTCAE) version 5.0, and serious adverse events were defined as grade 3 or higher.

#### Ex vivo MFGI and quantitative spectroscopy

Back-table MFGI of the fresh lymph node specimen was performed with an IVIS Spectrum imaging system with the IVIS Lumina II imaging system as a backup system (PerkinElmer, Waltham, USA). Quantitative multidiameter single-fiber reflectance and single-fiber fluorescence (MDSFR/SFF) spectroscopy measurements of fresh PTC nodal metastases, normal lymph nodes, and fat/connective tissue were performed in triplicates to correct for the optical tissue properties. This resulted in the intrinsic fluorescence value (Q.µ_a_^f^), as described previously [26]. Following formalin fixation and lymph node grossing, MFGI and spectroscopy were repeated. Finally, all tissue was embedded in paraffin according to standard pathological procedures. Details about image acquisition, quantitative spectroscopy, and data analysis are provided in the supplementary information.

#### Interim analysis

Following the initial inclusion of nine patients, an interim safety analysis was performed to evaluate outcome measures and was reported to the Data Safety Monitoring Board. The protocol allowed extending one or more cohorts with three patients per cohort to acquire sufficient data points to select the dosage group with optimal tumor-to-background ratio (TBR). After the dose extension, a second interim analysis was conducted to determine the optimal dose. The optimal dosage cohort was then extended to ten patients.

### Tracer binding validation

To assess MET expression status, 4-μm slides of PTC nodal metastases and normal lymph nodes of the three initial patients included in each dosage cohort were immunohistochemically stained (MET, hematoxylin and eosin). To evaluate microscopic EMI-137 tracer distribution, fluorescence microscopy was performed on representative 10-μm slides of PTC nodal metastases and normal lymph node tissue of three patients.

### Statistical analysis

A Welch *t*-test was used to detect significant differences between FGmRNA-signals of primary PTC and normal thyroid tissue. A multivariate permutation test was performed to control for multiple testing. This enabled us to detect differentially expressed genes with a false discovery rate of 5% at a confidence level of 95%. Normally distributed data are presented with mean values, standard deviations (SD), and a Student’s *t*-test was used to test for significance. Non-normally distributed data are presented as median with interquartile range (IQR), and Wilcoxon (paired data) or Fisher exact and Mann–Whitney *U* tests (independent data) and associated post hoc testing were used to compare groups. Kaplan–Meier survival analysis with log-rank tests was performed to compare LRFS and OS per MET staining status. Multivariate analysis using Cox proportional-hazards model was performed to identify factors associated with locoregional recurrence. Receiver-operator curves (ROCs) were used to calculate a device-specific threshold median fluorescent intensity for PTC nodal metastases in the optimal dosage cohort. With this device-specific threshold, the diagnostic accuracy of MFGI for the detection of positive lymph node levels was calculated (supplementary information). If one or more lymph nodes within a level had a fluorescence intensity above the device-specific threshold, this was defined as a fluorescence-positive level. A *p* value < 0.05 was regarded as significant. Statistics and graph design were performed using SPSS (version 25, IBM, Armonk, NY, USA) and GraphPad Prism (version 9.0, GraphPad Software Inc, San Diego, CA, USA).

## Results

### Target selection

To determine the number of upregulated genes in PTC, class comparison between the FGmRNA-profiles of 97 primary PTCs and 80 normal thyroid samples was performed. This comparison revealed 1702 significantly upregulated genes in PTC (supplementary table 2). Within this set of genes, we identified five genes targeted by one of 12 clinical available NIRF tracers: *MET* (ranked 11; tumor-specific target of the clinically available NIRF tracer EMI-137), *PSMA 7* and *PSMA 1* (ranked 685 and 1070; targets of MDX1201-A488), and *CTSK* and *CTSH* (ranked 668 and 1672; targets of LUM015).

*MET* was selected for immunohistochemical assessment of protein expression in primary PTC tissue and normal thyroid tissue based on its ranking. By quantifying the MET staining intensity of TMA cores, we found that the *H*-score of primary PTC tissue from 741 patients was significantly higher when compared to non-paired normal thyroid tissue from 108 patients (200.0 [IQR 150.0–258.0] versus 68.0 [IQR 20.0–100.0]; *p* < 0.0001). Of the primary PTCs, 559 out of 741 (75.4%) stained positive for MET, while positive staining was observed in only three out of 108 (2.8%) of normal thyroid tissue specimens. Further investigation of paired primary PTC and adjacent normal thyroid tissue from 70 patients confirmed the higher MET *H*-score in PTC (200.0 [IQR 100.0–254.0] versus 63.0 [IQR 11.0–100.0]; *p* < 0.0001).

**Fig. 2:**
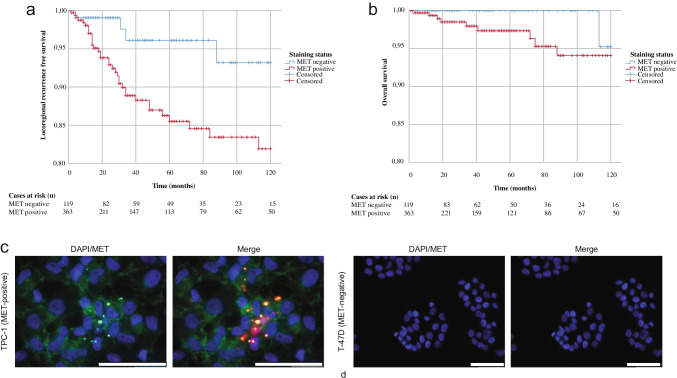
In vitro specific binding analysis of EMI-137 and association of MET immunohistochemical staining intensities with oncological outcome parameters in PTC. (**a**) The 10-year locoregional recurrence-free survival for patients with positive (red line) and negative (blue line) MET staining status is depicted. Patients with a positive staining status have worse 10-year locoregional recurrence-free survival rates compared to patients with negative staining (*p* = 0.017). (**b**) The similar 10-year overall survival between both groups (*p* = 0.21). Immunofluorescent staining of MET-positive (TPC-1, c) and MET-negative (T-47D, d) cell lines, with nuclei (blue), MET (green), and EMI-137 (red); scale bar is 50 µm; abbreviation: PTC, papillary thyroid cancer

In the next step, MET staining intensity was associated with clinical data. LRFS and OS data were available for 484 out 741 patients (65.3%) with primary PTC tissue who were included in the TMA (Table 1) with a median follow-up of 36.0 (IQR 13.0–81.8) months. Patients with positive MET staining of the primary PTC tumor (*n* = 364) were more frequently diagnosed with pT3 or pT4 PTC (40.7% versus 28.3%; *p* = 0.02), nodal metastases (42.4% versus 25.9%; *p* = 0.005), extrathyroidal extension (38.2% versus 21.7%; *p* = 0.001), and a positive BRAF^V600E^ status (73.4% versus 22.5%; *p* < 0.0001). Out of 484 patients, locoregional recurrence or death occurred within 10 years from diagnosis in 39 patients (8.1%) and 10 patients (2.1%), respectively. The estimated 10-year LRFS and OS of the entire cohort were 84.5% and 94.5%, respectively. Patients with PTC and positive MET staining had a worse 10-year LRFS compared to patients with negative staining (81.9% versus 93.2%; *p* = 0.017; Fig. 2a), whereas the difference in 10-year OS was not significant (94.1% versus 95.2%; *p* = 0.21; Fig. 2b). Following univariate and multiple regression analysis, a positive MET expression status (HR 4.76 [95% C.I. 1.14–19.90]; *p* = 0.03) and extrathyroidal extension (HR 4.95 [95% C.I. 2.10–11.69]; *p* ≤ 0.0001) were the only factors associated with locoregional recurrence in PTC (supplementary table 4).

**Table 1:** Characteristics of patients with available follow-up data who were included in the TMA for the target selection process

	Positive MET staining (*n* = 364)	Negative MET staining (*n* = 120)	Total (*n* = 484)	*p* value
*General characteristics*				
Gender—no. (%)				0.22
* Male*	85 (23.4)	35 (29.2)	120 (24.8)	
* Female*	279 (76.6)	85 (70.8)	364 (75.2)	
Age, years—mean (SD)	46.8 (15.9)	46.8 (16.0)	46.8 (15.9)	0.97
*Tumor characteristics*				
T-status—no. (%)				0.022
* pT1*	152 (41.8)	61 (50.8)	213 (44.0)	
* pT2*	62 (17.0)	23 (19.2)	85 (17.6)	
* pT3*	131 (36.0)	31 (25.8)	162 (33.5)	
* pT4*	17 (4.7)	3 (2.5)	20 (4.1)	
* Missing*	2 (0.5)	2 (1.7)	4 (0.8)	
N-stage—no. (%)				0.005
* cN0/pN0*	209 (57.4)	88 (73.3)	135 (27.9)	
* pN1a*	89 (24.5)	14 (11.7)	103 (21.3)	
* pN1b*	65 (17.9)	17 (14.2)	82 (16.9)	
* Missing*	2 (0.5)	1 (0.8)	2 (0.4)	
M-stage—no. (%)				0.69
* M0*	354 (97.3)	117 (97.5)	471 (97.3)	
* M1*	7 (1.9)	1 (0.8)	8 (1.7)	
* Missing*	3 (0.8)	2 (1.7)	5 (1.0)	
Tumor size (mm)—median (IQR)	18.0 (10.0–30.0)	17.0 (9.8–35.0)	18.0 (10.0–30.0)	0.83
Multifocality—no. (%)				0.51
* Yes*	160 (44.0)	52 (43.3)	270 (55.8)	
* No*	203 (55.8)	67 (55.8)	212 (43.8)	
* Missing*	3 (0.8)	1 (0.8)	2 (0.4)	
Vascular invasion—no. (%)				0.16
* Present*	109 (29.9)	29 (24.2)	341 (70.5)	
* Absent*	253 (69.5)	88 (73.3)	138 (28.5)	
* Missing*	2 (0.5)	3 (2.5)	5 (1.0)	
Extrathyroidal extension—no. (%)				
* Present*	139 (38.2)	26 (21.7)	315 (65.1)	0.001
* Absent*	223 (61.3)	92 (76.6)	165 (34.1)	
* Missing*	2 (0.5)	2 (1.7)	4 (0.8)	
BRAFV600E status—no. (%)				< 0.0001
* Positive*	267 (73.4)	27 (22.5)	294 (60.7)	
* Negative*	97 (26.6)	93 (77.5)	190 (39.3)	
*Treatment characteristics*				
Lymph node dissection—no. (%)				0.009
* Yes*	233 (64.0)	60 (50.0)	293 (60.6)	
* No*	130 (35.7)	59 (49.2)	189 (39.0)	
* Missing*	1 (0.3)	1 (0.8)	2 (0.4)	
Radioactive iodine treatment—no. (%)				0.083
* Yes*	319 (87.6)	95 (79.2)	414 (85.6)	
* No*	42 (11.6)	21 (17.5)	63 (13.0)	
* Missing*	3 (0.8)	4 (3.3)	7 (1.4)	
* Dosage (GBq)*—*median (IQR)*	4.5 (3.9–6.0)	4.3 (2.7–6.0)	4.5 (3.8–6.0)	0.084

Abbreviations: *IQR*, interquartile range; *N-stage*, nodal stage; *M-stage*, metastatic stage; *GBq*, gigabecquerel; *SD*, standard deviation; *T*, tumor stage

The specific binding of its target ligand EMI-137 was confirmed with in vitro studies, which showed cytoplasmatic accumulation of EMI-137 in the MET-positive PTC cell line (TPC1; Fig. 2c), whereas no EMI-137 binding was observed in the MET-negative breast cancer cell line (T-47D; Fig. 2d).

### Clinical tracer safety assessment and optimal dosage selection

#### Patient characteristics

We included 19 patients (UMCG *n* = 18; EMC *n* = 1) in the dose-escalation study (Table 2). Patients were treated with a CLND (*n* = 8), unilateral or bilateral lymph node dissection including CLND (*n* = 7), or selective lymph node dissection (*n* = 4). Histopathological review confirmed the presence of PTC nodal metastases in 15 of these patients.

**Table 2:** Characteristics of patients included in the phase 1 dose-escalation study per dosage cohort

	0.09 mg/kg (*n* = 3)	0.13 mg/kg (*n* = 10)	0.18 mg/kg (*n* = 6)	Total (*n* = 19)
*General characteristics*				
Female sex—no. (%)	1 (33.3)	6 (60.0)	3 (50.0)	10 (52.6)
Male sex—no. (%)	2 (66.7)	4 (40.0)	3 (50.0)	9 (47.4)
Age, years—median (IQR)	70.0 (69.0–n/a)	58.5 (46.8–72.3)	52.5 (32.5–66.8)	62.0 (46.0–72.0)
Weight, kg—median (IQR)	84.0 (77.0–n/a)	89.0 (77.3–100.8)	75.0 (69.3–85.6)	84.0 (75.0–94.0)
*Indication*				
First presentation—no. (%)	1 (33.3)	8 (80.0)	6 (100)	15 (78.9)
Recurrent disease—no. (%)	2 (66.6)	2 (20.0)	0	4 (21.1)
*Thyroid surgery*				
None—no. (%)	2 (66.6)	2 (20.0)	0	4 (21.1)
Hemithyroidectomy—no. (%)	0	1 (10.0)	1 (16.7)	2 (10.5)
Total thyroidectomy—no. (%)	1 (33.3)	7 (70.0)	5 (83.3)	13 (68.4)
*Lymph node dissections*				
CLND				
None—no. (%)	1 (33.3)	1 (10.0)	0	2 (10.5)
Unilateral—no. (%)	1 (33.3)	1 (10.0)	3 (50.0)	5 (26.3)
Bilateral—no. (%)	1 (33.3)	8 (80.0)	3 (50.0)	12 (63.2)
Lateral neck dissection				
None—no. (%)	2 (66.7)	4 (40.0)	4 (66.7)	10 (52.6)
Selective unilateral—no. (%)	1 (33.3)	1 (10.0)	0	2 (10.5)
Unilateral—no. (%)	0	3 (30.0)	1 (16.6)	4 (21.1)
Bilateral—no. (%)	0	2 (20.0)	1 (16.6)	3 (15.8)
*T-stage*				
pT1—no. (%)	1 (33.3)	3 (30.0)	2 (33.3)	6 (31.6)
pT2—no. (%)	0	0	4 (66.7)	4 (21.1)
pT3—no. (%)	0	3 (30.0)	0	3 (15.8)
pT4—no. (%)	0	2 (20.0)	0	2 (10.5)
Not applicable – no. (%)	2 (66.7)	2 (20.0)	0	4 (21.1)
*N-stage*				
pN0—no. (%)	1 (33.3)	1 (10.0)	2 (33.3)	4 (21.1)
pN1a—no. (%)	1 (33.3)	3 (30.0)	2 (33.3)	6 (31.6)
pN1b—no. (%)	1 (33.3)	6 (60.0)	2 (33.3)	9 (47.4)
*Grossed lymph nodes*				
PTC nodal metastases—no. (%)	3 (21.4)	41 (14.2)	32 (28.3)	76 (18.3)
Normal lymph nodes—no. (%)	11 (78.6)	248 (85.8)	81 (71.7)	340 (81.7)

Staging was performed according to the 8th edition of the American Joint Committee on Cancer staging system. Abbreviations: *CLND*, central compartment lymph node dissection; *IQR*, interquartile range; *N-stage*, nodal stage; *PTC*, papillary thyroid cancer; *T-stage*, tumor stage

#### Perioperative intravenous administration of EMI-137 is safe

No serious adverse events related to the administration of EMI-137 occurred. One grade 1 adverse event was possibly related to EMI-137 administration, consisting of a spontaneously resolved episode of flushing 25 min after EMI-137 administration in the 0.18 mg/kg dosage cohort.

#### A dosage of 0.13 mg/kg EMI-137 found to be optimal for PTC nodal metastasis detection using MFGI

Patients received 0.09 mg/kg (*n* = 3), 0.13 mg/kg (*n* = 10), and 0.18 mg/kg (*n* = 6) EMI-137 intravenously. Three out of four patients included in the 0.13 mg/kg dose extension cohort after the second interim analysis were imaged with the IVIS Lumina II due to a technical failure of the IVIS Spectrum. After lymph node grossing, 76 PTC nodal metastases and 340 normal lymph nodes were assessed with fluorescence epi-illumination imaging. A detailed description of the number of imaged lymph nodes per dosage cohort is provided in Table 2. No TBR was calculated for 7 out of 19 patients due to the absence of either PTC nodal metastases (*n* = 4) or normal lymph nodes (*n* = 3).

**Fig. 3:**
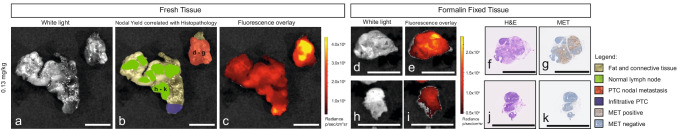
Representative images from PTC nodal metastases and normal lymph nodes imaged with the IVIS Spectrum in the 0.13 mg/kg dosage cohort. MFGI images from the fresh nodal dissection specimen and representative formalin-fixed PTC nodal metastasis (**d–g**) and normal lymph node (**h–k**) are presented. The exact location of the grossed lymph nodes is provided and correlated to final histopathology (**b**). Fluorescence intensities for the formalin-fixed PTC nodal metastases and normal lymph nodes are scaled. The scale is provided in radiance. Corresponding H&E (**f** and **j**) and MET stained slides (**g** and **k**) are provided per presented lymph node. Scale bars represent 10 mm

**Fig. 4:**
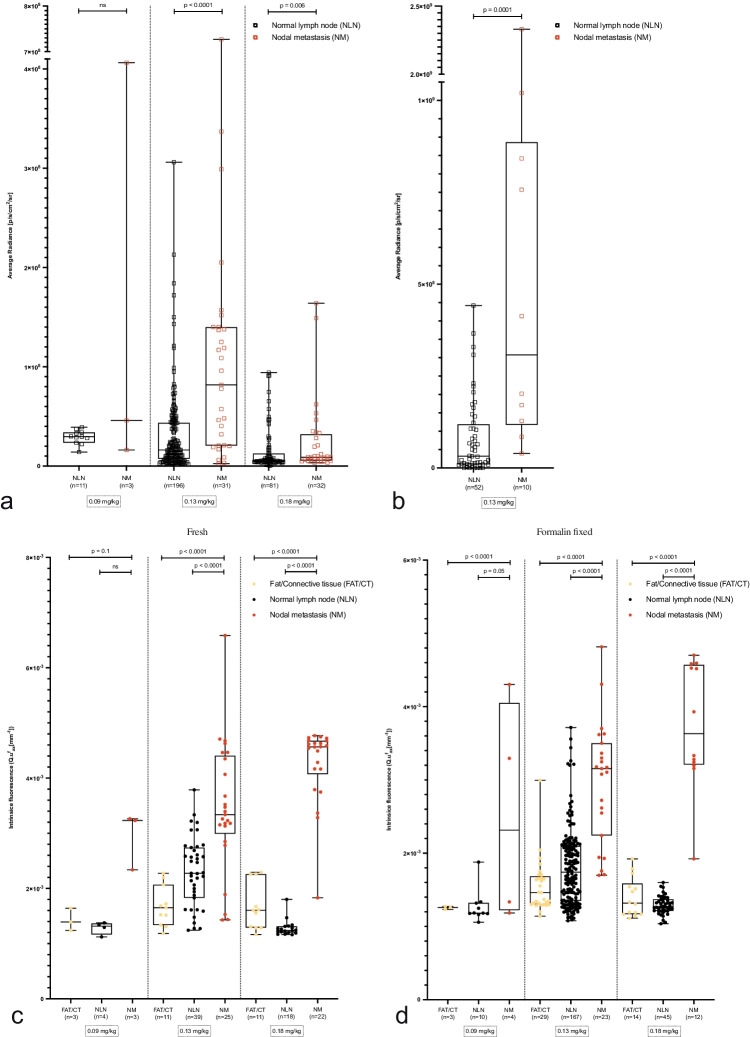
(**a**) An overview of fluorescent intensities per dosage cohort of grossed formalin-fixed PTC nodal metastases and normal lymph nodes imaged with the IVIS Spectrum. (**b**) The fluorescence intensities of PTC nodal metastases and normal lymph nodes of patients in the 0.13 mg/kg dosage cohort imaged using the IVIS Lumina. An overview of the intrinsic fluorescence measured with quantitative spectroscopy in fresh (**c**) and formalin-fixed (**d**) PTC nodal metastases and normal lymph nodes, respectively. Abbreviations: CT, connective tissue; NLN, normal lymph node; NM, nodal metastases; PTC, papillary thyroid cancer

Representative IVIS Spectrum images are shown in Fig. 3 and supplementary Fig. 2. Quantification of IVIS Spectrum images confirmed higher median fluorescence signals in PTC nodal metastases compared to normal lymph nodes in the 0.13 mg/kg (*p* < 0.0001) and 0.18 mg/kg (*p* = 0.006) dosage cohorts (Fig. 4a and Table 3). Data from the IVIS Lumina II acquired from three patients in the 0.13 mg/kg dose extension cohort confirmed the higher median fluorescence signal in PTC nodal metastases (*p* < 0.0001; Fig. 4b and Table 3). A median TBR of 3.1 (IQR 3.4) was observed in the 0.13 mg/kg cohort, compared to 1.3 (IQR 0.5) and 1.5 (IQR 1.8) in the 0.09 mg/kg and 0.18 mg/kg cohorts (supplementary Fig. 3).

**Table 3:** MFGI and MDSFR/SFF spectroscopy quantification values of 0.13 mg/kg and 0.18 mg/kg dosage cohorts

	0.13 mg/kg	0.18 mg/kg
*MFGI, p/s/cm*^*2*^*/sr—median (IQR)*		
IVIS Spectrum		
Grossed PTC nodal metastases	8.2 × 10^7^ (1.2 × 10^8^)	4.6 × 10^7^ (2.7 × 10^7^)
Grossed normal lymph nodes	1.6 × 10^7^ (3.6 × 10^7^)	3.0 × 10^7^ (8.6 × 10^6^)
IVIS Illumina		
Grossed PTC nodal metastases	3.1 × 10^8^ (7.7 × 10^8^)	n/a
Grossed normal lymph nodes	2.4 × 10^7^ (9.9 × 10^7^)	n/a
*Spectroscopy, Q.µ*_*a*_^*f*^_*ax*_*mm*^*−1*^—*median (IQR)*		
Fresh PTC nodal metastases	3.3 × 10^−3^ (1.4 × 10^−3^)	3.6 × 10^−3^ (1.4 × 10^−3^)
Fresh normal lymph nodes	2.3 × 10^−3^ (9.1 × 10^−4^)	1.3 × 10^−3^ (1.6 × 10^−4^)
Fresh fat/connective tissue	1.7 × 10^−3^ (7.3 × 10^−4^)	1.3 × 10^−3^ (4.2 × 10^−4^)
Grossed PTC nodal metastases	3.2 × 10^−3^ (1.3 × 10^−3^)	3.2 × 10^−3^ (1.3 × 10^−3^)
Grossed normal lymph nodes	1.7 × 10^−3^ (7.5 × 10^−4^)	1.7 × 10^−3^ (7.5 × 10^−3^)
Grossed fat/connective tissue	1.5 × 10^−3^ (3.9 × 10^−4^)	1.5 × 10^−3^ (3.9 × 10^−4^)

The median radiance (p/s/cm^2^/sr) and median intrinsic fluorescence (Q.µ_a_^f^_ax_mm^−1^) calculated from MFGI and MDSFR/SFF spectroscopy, respectively, are provided for the dosage cohorts with a significant difference in the quantified values between PTC nodal metastases and normal lymph nodes. MFGI was performed in formalin-fixed grossed lymph nodes, whereas MDSFR/SFF spectroscopy was performed in both fresh nodal dissection specimens and formalin-fixed grossed lymph nodes. Abbreviations: *cm*, centimeter; *IQR*, interquartile range; *MDSFR/SFF*, multidiameter single-fiber reflectance and single-fiber fluorescence; *MFGI*, molecular fluorescence-guided imaging; *mm*, millimeter; *p*, photons; *PTC*, papillary thyroid cancer; *s*, second; *sr*, steradian; *kg*, kilogram

To estimate the diagnostic accuracy of MFGI in the optimal dosage cohort, we first determined device-specific threshold median fluorescent intensities for the detection of PTC nodal metastases at 1.7 × 10^7^ p/s/cm^2^/sr for the IVIS Spectrum (supplementary Fig. 4a) and 8.3 × 10^7^ p/s/cm^2^/sr for the IVIS Illumina II (supplementary Fig. 4b). Using these device-specific thresholds, we calculated a level-specific sensitivity of 87.5%, a specificity of 26.3%, and an NPV of 83.3% to detect levels containing PTC-positive lymph nodes with MFGI. We identified 17 out of 18 (94.4%) levels with PTC nodal metastases in the patients (*n* = 10) in the 0.13 mg/kg dosage cohort as true positive. In contrast, five out of 19 levels (26.3%) without nodal metastases were true negative (supplementary table 3).

#### MDSFR/SFF spectroscopy detected PTC nodal metastases

We performed quantitative spectroscopy in fresh tissue from 16 patients to assess the intrinsic fluorescence of 50 PTC nodal metastases, 61 normal lymph nodes, and 25 fat/connective tissue specimens. Compared to normal lymph nodes, a higher intrinsic fluorescence of PTC nodal metastases (*p* < 0.0001) and fat/connective tissue (*p* < 0.0001) was found in the 0.13 mg/kg and 0.18 mg/kg dosage cohorts (Fig. 4c and Table 3). Quantitative spectroscopy data on formalin-fixed tissue of 39 PTC nodal metastases, 222 normal lymph nodes, and 46 fat/connective tissue specimens acquired from 16 patients confirmed a higher intrinsic fluorescent value in PTC nodal metastases compared to normal lymph nodes (*p* < 0.0001) and fat/connective tissue (*p* < 0.0001) in the 0.13 mg/kg and 0.18 mg/kg dosage cohorts (Fig. 4d and Table 3).

### Tracer binding validation

#### PTC nodal metastases shown to have MET overexpression and cytoplasmatic accumulation of EMI-137

A positive MET staining status was observed frequently in PTC nodal metastases, but was nearly absent in normal lymph nodes (81.3% [*n* = 16] versus 1.4% [*n* = 74]; *p* < 0.0001). Cross-sectional images made with confocal fluorescent microscopy confirmed a clear cytoplasmic EMI-137 signal in PTC nodal metastases. In contrast, the cytoplasmic concentration of EMI-137 in normal lymph nodes without MET expression was low (supplementary Fig. 5a-d).

## Discussion

This international multicenter translational fluorescence-guided study demonstrates that detection of PTC nodal metastases using MFGI and quantitative spectroscopy after preoperative intravenous administration of EMI-137 is feasible. During the target selection process, we found that MET was overexpressed in primary PTC compared to normal thyroid tissue at both the mRNA and protein level. An increased risk of 10-year locoregional disease recurrence was found in patients with positive MET expression of primary PTC. Together with extrathyroidal extension, a positive MET expression status was associated with an increased locoregional recurrence risk. We also determined that it is safe to administer EMI-137 to patients with PTC undergoing surgery and that MFGI could reduce the number of negative prophylactic CLNDs by 26.3%.

Previous studies that performed immunohistochemistry or gene expression analysis—usually in smaller cohorts—reported MET overexpression in primary PTC at both gene and protein level compared to normal thyroid tissue [17, 27–39]. Our results were in line with the small number of studies that associated MET expression status of primary PTC with clinical characteristics and oncological outcomes. They reported that patients with MET-positive PTC have increased extrathyroidal extension rates, higher tumor stages, and more frequent nodal metastases [27, 34–36, 38]. However, our study also revealed that MET is overexpressed in PTC nodal metastases compared to normal lymph nodes. A second novel finding from our study concerns the higher prevalence of BRAF^V600E^ mutations and an increased 10-year locoregional recurrence rate in patients with MET-positive PTC, which suggests that that MET positivity is associated with a biologically more aggressive PTC variant. MET overexpression could thus be useful to identify patients with MET-positive PTC who may benefit more from alternative treatment schemes or intensified follow-up than patients with MET-negative PTC.

EMI-137 has been tested previously for its safety and feasibility to improve colorectal polyp detection during fluorescence-guided endoscopy procedures [25, 40]. The pharmacokinetics of the selected NIRF tracer EMI-137 enable a variable injection-imaging interval ranging between 1 and 3 h. [40] This greatly increases its practical and logistical applicability in a surgical setting compared to other clinically available fluorescent-labeled antibodies that need to be administered multiple days before surgery [7]. Finally, our study has shown that perioperative administration of EMI-137 in patients with PTC is safe and that subsequent MFGI and quantitative spectroscopy is feasible to detect PTC nodal metastases.

The ability of molecular imaging to differentiate PTC nodal metastases from benign lymph nodes is dependent on lymphatic biodistribution and specific binding of EMI-137 following intravenous injection. We have shown that EMI-137 accumulates within the cytoplasm of the TPC1 cell line and PTC nodal metastases, suggesting internalization of the NIRF tracer in MET-positive cells. The cytoplasmatic localization of EMI-137 in normal lymph nodes was much lower, possibly due to a minimal presence of the MET receptor or passive drainage of EMI-137 via the lymphatic system. Our data suggest that EMI-137 circulates through the lymphatic system and eventually binds specifically with MET*-*expressing PTC tumor cells.

Our study has several limitations. First, target validation was based on tissue acquired by biopsy, which may not fully reflect tumor heterogeneity. Second, the 675-nm emission wavelength of EMI-137 may complicate MFGI because it interferes with background autofluorescence and has shallower penetration depth. However, dyes emitting at this wavelength tend to be brighter than their higher wavelength counterparts. In our study, MFGI resulted in a TBR of 3.1 in the optimal dosage cohort. Our quantitative spectroscopy data showed that the intrinsic fluorescence of EMI-137 is sufficient to detect PTC nodal metastases in fresh nodal dissection specimens. Important strengths of our study include the multiple-step target selection process, the validation of MET expression with the largest PTC TMA cohort to date (741 patients), and the multicenter design. This latter aspect supports the feasibility of implementing the infrastructure required for MFGI and quantitative spectroscopy in multiple centers.

We found that MFGI may reduce the number of negative prophylactic CLNDs by more than a quarter. By correcting for scattering and absorption, spectroscopy may reduce the number of false-positive levels. Complementing MFGI with spectroscopy could improve the detection of true negative central compartments even more. Additional studies are needed to determine the actual diagnostic accuracy of EMI-137 targeted imaging and explore the clinical implications of MET overexpression in patients with PTC who are undergoing surgery. Clinical translation of MFGI and quantitative spectroscopy for detection of PTC nodal metastases requires a phase 2 study to assess the intraoperative in vivo diagnostic accuracy of both modalities after intravenous injection with EMI-137. Our study may pave the way for the translation of MET targeted MFGI and spectroscopy for the intraoperative visualization of other tumors with MET overexpression, such as head and neck, colon, breast, pancreatic, and penile cancer.

In conclusion, MET is significantly overexpressed in both primary PTC and nodal metastases. It can be used to identify biologically more aggressive variants that might require alternative treatment or follow-up. EMI-137 can be used safely in the perioperative setting, and in combination with MFGI and quantitative spectroscopy could improve intraoperative lymph node staging and potentially reduce the 50% true negative PLCND with more than 25%. This could improve the selection of patients that benefit from omitting a prophylactic CLND, ultimately reducing overtreatment and associated morbidity in PTC management.

## Supplementary Information

Below is the link to the electronic supplementary material.

Supplementary file1 (DOCX 40019 KB)

## Data Availability

The study protocol and deidentified data used for this study is available upon request via the corresponding author after approval of all authors, review of a proposal, and following establishment of data transfer agreements.
