# Supplementary material for: Intraoperative MET-receptor targeted fluorescent imaging and spectroscopy for lymph node detection in papillary thyroid cancer: novel diagnostic tools for more selective central lymph node compartment dissection
Source: Eur J Nucl Med Mol Imaging. 2022 Apr 7;49(10):3557–70. doi: 10.1007/s00259-022-05763-3 (PMC9308606; doi:10.1007/s00259-022-05763-3)
Supplement: Supplementary file 1 — Supplementary file1 (DOCX 40019 KB) [file 259_2022_5763_MOESM1_ESM.docx]

**Supplementary information**

**Methods**

*Microarray expression data acquisition and processing*

Publicly available microarray expression data of primary PTCs and normal thyroid samples generated with the Affymetrix HG-U133A (GPL96) and Affymetrix HG-U133 Plus 2.0 (GPL570) platforms were extracted from the Gene Expression Omnibus (GEO)^1^ on 11-05-2015. Detailed information on the search query, manual curation, duplicate removal, preprocessing, and quality control has been described previously.^2^ Primary PTC and normal thyroid tissue acquired from patients with known exposure to radiation were excluded from the analysis. After manual curation, duplicate removal, and quality control, 97 PTC and 80 normal thyroid samples were included for the generation of FGmRNA-profiles. GEO accession identifiers and metadata of these samples are provided in Supplementary Table 1.

*Tissue microarray: Immunohistochemical staining scoring of staining intensities*

A monoclonal rabbit primary antibody (Cell Signaling Technology Cat# 8198, Danvers, United States RRID:AB_10858224) targeting the endogenous carboxy terminus of human MET was used for immunohistochemical staining. Immunohistochemical staining was performed at the Department of Pathology of University Medical Center Utrecht (UMCU), using a standardized protocol (Ventana BenchMark ULTRA, Roche Holding AG, Basel, Switzerland) of a tissue microarray (TMA) with primary PTC tumor tissue of 741 patients and normal thyroid tissue of 108 patients. For each patient, two or more tissue cores with a diameter of one millimeter were present on the TMA. When cores contained both PTC and adjacent normal thyroid tissue, each histotype was scored individually and regarded as a separate core. An expert pathologist determined staining intensity at 10x to 20x magnification. Per patient an H-score ranging between 0 and 300 was calculated from available cores based on the percentage of cells (0% to 100%) for each staining intensity (0, 1+, 2+, 3+) in each core using the formula [1 x (% cells 1+) + 2 x (% cells 2+) + 3 x (% cells 3+)]. In this way, a H-score per tissue type per patient was calculated. The H-scores were ranked as follows: 0 to 49 (staining intensity 0; negative), 50 to 149 (staining intensity 1+; low), 150 to 249 (staining intensity 2+; marked), 250 to 300 (staining intensity 3+, strong). An H-score ≥ 150 (equivalent to an average staining intensity ≥2+) was defined as positive staining. An overview of representative images of each staining intensity for both PTC primary tumor tissue and normal thyroid tissue is provided in Supplementary Figure 1.

*Acquisition of clinical data of patients with tissue included in the tissue microarray*

Following approval of the Northern Sydney Local Health District Human Research Ethics Committee, the prospective database of the Royal North Shore Unit of Endocrine Surgery was accessed to acquire clinical follow-up data for patients with tissue included in the TMA. For patients with available follow-up data, demographics, tumor characteristics (including BRAF^V600E^ status) and treatment details were acquired. The protocol for BRAF^V600E^ immunohistochemical staining and scoring has been described previously.^3^ Recurrent disease was defined as structural locoregional disease identified with imaging (radioactive iodine scans, ultrasound, MRI or CT) or histopathological confirmation by cytology, biopsy or resection. OS was defined as time from initial diagnosis to final follow-up or death.

*In vitro cell-line-specific binding analysis*

Human MET*-*positive (PTC, TPC1, RRID:CVCL_6298) and MET*-*negative cell lines (breast cancer, T47D, RRID:CVCL_0553) were cultured in RPMI-1640 medium supplemented with 10% fetal calf serum (Thermo Scientific) and Pen/Strep (Invitrogen). Positive and negative MET expression status in TPC1 and T47D cell lines was confirmed by quantitative Polymerase Chain Reaction (qPCR, data not shown). For immunofluorescence analysis, the MET antibody (1:300, CST) was used to detect the expression and localization of MET. Cells were plated onto gelatin-coated glass coverslips and allowed to attach overnight. The attached cells were exposed to 0.038 mg/mL EMI-137 in complete growth medium for 30 minutes before washing with PBS, PFA fixation, permeabilization and incubation with primary antibody. After incubation with secondary antibodies, the coverslips were mounted using ProLong™ Glass Antifade Mountant containing NucBlue™ Stain (Thermo Fisher) and imaged with a Leica SP8X DLS confocal microscope (Leica Biosystems GmbH, Wetzlar, Germany).

*Ex vivo imaging settings*

The stage temperature of the IVIS imaging systems was set to 20 degrees prior to imaging to prevent the fresh and formalin-fixed tissue from drying. Epi-fluorescence images with the IVIS Spectrum were taken using excitation/emission filter settings of 640/680 nm, an exposure of 2 seconds and binning 8. For the IVIS Lumina II, excitation/emission filter settings of 640/595-770, a 2 second exposure and binning of 4 was used to acquire images of the tissue. Both fresh and formalin-fixed tissue were imaged from front and back.

*Fluorescence quantification and target-to-background calculations*

LivingImage (version 4.3.1) was used to draw regions of interest (ROI) on monochrome IVIS images of formalin-fixed grossed PTC nodal metastases and normal lymph nodes, which were assessed as such by final histopathology. The median fluorescence intensity (p/sec/cm^2^/sr) with interquartile range (IQR) was calculated per ROI from the fluorescent overlay image. The target-to-background ratio was calculated per patient by dividing the median fluorescence intensity (MFI) of nodal metastases by the MFI of normal lymph nodes. The diagnostic accuracy of MFGI in the optimal dosage cohort was assessed per level by correlating median fluorescence intensities with histopathology. A level was defined as fluorescence-positive if one or more of the imaged lymph nodes within a level had a cut-off median fluorescence intensity at or above a threshold based on a receiver operator curve (ROC) for both IVIS Spectrum and IVIS Lumina II. To maximize the detection of true- negative levels, the receiver-operator curve (ROC) threshold was based on the optimal sensitivity for the detection of nodal metastases. Each MDSFR/SFF spectroscopy measurement taken in fresh and formalin-fixed tissue was correlated with final histopathology. Spectroscopy measurements were performed in triplicate, and a weighted mean Q.µ_a_^f^ was calculated using the fit confidence intervals of individual measurements. ^4^ The weighted means Q.µ_a_^f^ of fresh and formalin-fixed PTC nodal metastases and normal lymph nodes were divided to calculate the tumor-to-background ratio per patient.

*Immunohistochemistry of nodal metastases*

To assess the MET expression status of PTC nodal metastases and normal lymph nodes, two back-to-back 4μm slides were acquired of formalin-fixed, paraffin-embedded (FFPE) blocks of all grossed PTC nodal metastases and normal lymph nodes of the initial nine patients included in the dose escalation study (i.e., three patients per dosage cohort). MET staining was performed at the Department of Pathology of the UMCU with the same protocol used for immunohistochemical staining of the TMA. Haematoxylin & eosin staining was performed using standard protocols of the Department of Pathology of the University Medical Center Groningen. The MET staining intensity was scored by an expert pathologist and quantified as 0 (negative), 1+ (low), 2+ (marked) or 3+ (high). A staining intensity ≥2+ was defined as positive staining, which is in line with the cut-off value used for scoring the MET staining intensity of the TMA.

*Fluorescence microscopy*

To evaluate EMI-137 accumulation on a microscopic level in normal lymph nodes and PTC nodal metastases (as described previously ^5^ ), a Leica SP8X DLS confocal microscope (Leica Biosystems GmbH, Wetzlar, Germany) was used for fluorescence microscopy on one representative 10 μm slide per patient. Consistent settings across magnifications on the I (DAPI, nuclei) and Y5 (Cy5, corresponding to EMI-137 excitation and emission) filter cube were used.

**References**

1 Barrett T, Wilhite SE, Ledoux P, *et al.* NCBI GEO: archive for functional genomics data sets--update. *Nucleic Acids Res* 2013; **41**: D991–5.

2 Jonker PKC, van Dam GM, Oosting SF, Kruijff S, Fehrmann RSN. Identification of novel therapeutic targets in anaplastic thyroid carcinoma using functional genomic mRNA-profiling: Paving the way for new avenues? *Surgery* 2017; **161**: 202–11.

3 Fraser S, Go C, Aniss A, *et al.* BRAFV600E Mutation is Associated with Decreased Disease-Free Survival in Papillary Thyroid Cancer. *World J Surg* 2016; **40**: 1618–24.

4 Amelink A, Robinson DJ, Sterenborg HJCM. Confidence intervals on fit parameters derived from optical reflectance spectroscopy measurements. *J Biomed Opt* 2008; **13**: 054044.

5 Koller M, Qiu S-Q, Linssen MD, *et al.* Implementation and benchmarking of a novel analytical framework to clinically evaluate tumor-specific fluorescent tracers. *Nat Commun* 2018; **9**: 3739.

**Supplementary Tables**

**Supplementary table 3 – Diagnostic accuracy IVIS Spectrum and IVIS Lumina II in the optimal dosage cohort**

|  | True Negative levels (n) | False Positive levels (n) | True Positive  levels (n) | False Negative levels (n) |
| --- | --- | --- | --- | --- |
| Spectrum – n | 3 | 12 | 11 | 1 |
| Lumina II – n | 2 | 2 | 5 | 0 |
| Total – n | 5 | 14 | 17 | 1 |

Supplementary table 3 – Legend

For both IVIS Spectrum and IVIS Lumina the total number of imaged levels is provided. Fluorescence data were correlated to histopathology to assess diagnostic accuracy. A level was defined as fluorescence-positive if one or more of the imaged lymph nodes within a level had a cut-off median fluorescence intensity of 1.68x10^7^ p/sec/cm^2^/sr (IVIS Spectrum) or 8.23x10^7^ p/sec/cm^2^/sr (IVIS Lumina II).

**Supplementary table 4 – Factors associated with locoregional recurrence in papillary thyroid cancer**

|  | **Locoregional recurrence** | | | | |
| --- | --- | --- | --- | --- | --- |
|  | **Univariate** | | **Multivariate** | | |
| **Variable** | **HR (95% C.I.)** | **p-value** | **HR (95% C.I.)** | **p-value** |  |
| **MET expression status (positive vs negative)** | 3.29 (1.17 – 9.25) | 0.02 | 4.76 (1.14 – 19.90) | 0.03 |  |
| **Nodal status (pN1 vs pN0/cN0)** | 2.93 (1.54 – 5.59) | 0.001 | 1.38 (0.66 – 2.87) | 0.40 |  |
| **Tumor size (≥ 40 mm vs <40 mm)** | 2.11 (0.96 – 4.64) | 0.06 | N/A | N/A |  |
| **Multifocality (multifocal vs unifocal)** | 1.24 (0.65 – 2.36) | 0.51 | N/A | N/A |  |
| **Extrathyroidal extension (yes vs no)** | 7.58 (3.57 – 16.09) | <0.001 | 4.95 (2.10 – 11.69) | <0.001 |  |
| **Vascular invasion (yes vs no)** | 2.97 (1.54 – 5.72) | 0.001 | 1.49 (0.74 – 3.00) | 0.26 |  |

Supplementary table 4 – Legend

*C.I. = confidence interval; HR = Hazard Ratio; mm = millimeter*

Supplementary Figures

**Figure S1**

Representative images of staining intensities in primary PTC tissue. Overview of the 4 staining intensities observed as observed in primary PTC. L/N (H-score 0 to 49), 1+ (H-score 50 to 149), 2+ (H-score 150 to 249) and 3+ (H-score 250 to 300). An H-score ≥ 150 was considered positive.

**Figure S2**

Representative images from PTC nodal metastases and normal lymph nodes imaged with the IVIS Spectrum from all dosage cohort. MFGI images from the fresh nodal dissection specimen and representative formalin-fixed PTC nodal metastases and normal lymph nodes are shown. Fluorescence intensities for the formalin-fixed PTC nodal metastases and normal lymph nodes are scaled. The scale is provided in radiance. Scale bars represent 10 mm.

**Figure S3**

Box and whisker plot representing the tumor-to-background ratios per dosage cohort with median and all individual points.

**Figure S4**

Sensitivity and specificity of the NIRF tracer EMI-137. Figure 4a shows the receiver operators curve (ROC) for 31 PTC nodal metastases and 196 normal lymph nodes grossed from seven patients included in the 0.13 mg/kg optimal dosage cohort and imaged with the IVIS Spectrum. Figure 4b shows the ROC of 10 PTC nodal metastases and 52 normal lymph nodes grossed in the remaining three patients in the 0.13 mg/kg dosage cohort and imaged with the IVIS Lumina II. Grossed lymph nodes were imaged following overnight formalin fixation.

**Figure S5**

Fluorescence microscopy of PTC nodal metastases (a) and normal lymph node tissue (c) of patients who underwent EMI-137 administration with nuclei (Blue) and EMI-137 (Red). To assess MET expression status, immunohistochemical staining using 8191S was performed on a 4 µm slice acquired from the same PTC nodal metastases (b) and normal lymph node (d) as used for fluorescence microscopy. The scale bar represents 75 µm.

**D**

**C**

**B**

A
